# Modulation of 5' splice site selection using tailed oligonucleotides carrying splicing signals

**DOI:** 10.1186/1472-6750-6-5

**Published:** 2006-01-13

**Authors:** Daniel Gendron, Sandra Carriero, Daniel Garneau, Jonathan Villemaire, Roscoe Klinck, Sherif Abou Elela, Masad J Damha, Benoit Chabot

**Affiliations:** 1 Département de microbiologie et d'infectiologie, Faculté de médecine et des sciences de la santé, Université de Sherbrooke, Sherbrooke, Québec, Canada; 2 Department of chemistry, McGill University, Montréal, Québec, Canada

## Abstract

**Background:**

We previously described the use of tailed oligonucleotides as a means of reprogramming alternative pre-mRNA splicing in vitro and in vivo. The tailed oligonucleotides that were used interfere with splicing because they contain a portion complementary to sequences immediately upstream of the target 5' splice site combined with a non-hybridizing 5' tail carrying binding sites for the hnRNP A1/A2 proteins.

In the present study, we have tested the inhibitory activity of RNA oligonucleotides carrying different tail structures.

**Results:**

We show that an oligonucleotide with a 5' tail containing the human β-globin branch site sequence inhibits the use of the 5' splice site of Bcl-xL, albeit less efficiently than a tail containing binding sites for the hnRNP A1/A2 proteins. A branch site-containing tail positioned at the 3' end of the oligonucleotide also elicited splicing inhibition but not as efficiently as a 5' tail. The interfering activity of a 3' tail was improved by adding a 5' splice site sequence next to the branch site sequence. A 3' tail carrying a Y-shaped branch structure promoted similar splicing interference. The inclusion of branch site or 5' splice site sequences in the Y-shaped 3' tail further improved splicing inhibition.

**Conclusion:**

Our in vitro results indicate that a variety of tail architectures can be used to elicit splicing interference at low nanomolar concentrations, thereby broadening the scope and the potential impact of this antisense technology.

## Background

Alternative pre-messenger RNA splicing involves the differential use of splice sites, a process that represents a powerful and versatile way to control protein function. It is estimated that greater than 70% of the human genes may be alternatively spliced [1]. Striking examples highlighting the importance of alternative splicing are found in many systems including programmed cell death or apoptosis. Deregulation of apoptosis is often caused by alterations in the alternative splicing of regulatory genes including cell surface receptors such as Fas; mediators such as Bcl-2, Bcl-x and Bax; and members of the family of caspase proteases [2,3]. Bcl-x is alternatively spliced to produce Bcl-xL and Bcl-xS, two proteins with antagonistic function in apoptosis [4]. In cancers and cancer cell lines, the expression of the anti-apoptotic protein Bcl-xL is increased and overexpression of Bcl-xL is associated with decreased apoptosis, increased risk of metastasis, resistance to chemotherapeutic drugs and poor clinical outcome [5-9]. In contrast, the pro-apoptotic isoform Bcl-xS can sensitize cells to chemotherapeutic agents [9-13].

Given the pivotal role that alternative splicing plays in the diversification of protein function, strategies capable of specifically reprogramming alternative splicing should improve our ability to address the function of individual isoforms, and will provide tools to alter or correct aberrant splicing decisions in clinically relevant genes. Approaches that target alternative splicing can therefore be used to modulate the expression of protein isoforms with distinct activities. The annealing of oligonucleotides directly complementary to splice site sequences has been used to modulate splicing decisions of different pre-mRNAs including tau, dystrophin and the apoptotic regulator Bcl-x [reviewed in [14]]. For example, oligonucleotides complementary to the 5' splice site of Bcl-xL can shift splicing toward the pro-apoptotic Bcl-xS isoform, thereby increasing the sensitivity of the cells to chemotherapeutic agents [11]. Moreover, intraperitoneal injections of such oligonucleotides in mice can produce significant changes in the splicing of a target gene in several organs [15]. More recently, this strategy was used to prevent the use of cryptic splice sites in mutated lamin A transcripts responsible for Hutchinson-Gilford progeria syndrome [16]. However, directly targeting a 5' splice site can potentially create problems of specificity because 5' splice site sequences conform to a consensus. Thus, an oligonucleotide complementary to a selected 5' splice site may hybridize to off-target 5' splice sites, a situation that may compromise its effectiveness and blur the interpretation of the phenotype.

With the goal of improving the specificity and potency of oligonucleotide-based splicing inhibition, we have developed a strategy that relies on the use of an oligonucleotide complementary to exon sequences located immediately upstream of the target 5' splice site. The oligonucleotide also carries a non-hybridizing tail that contains binding sites for the hnRNP A1/A2 proteins [17]. The protein binding portion of the tail prevents U1 snRNP binding to the target 5' splice site and/or compromises spliceosome assembly. In extracts and in cells, this design inhibits splicing at the intended site more efficiently than an oligonucleotide directly targeting the 5' splice site, possibly because of reduced hybridization to secondary sites. Such bifunctional oligonucleotides are effective at nanomolar concentrations in cells, and are therefore unlikely to alter the availability of hnRNP A/B proteins which exist in micromolar amounts in actively dividing cells. Thus, positioning hnRNP A1/A2 proteins in the vicinity of a splice site using tailed oligonucleotides may be more specific and can be more effective than other antisense approaches.

Although hnRNP A1/A2 proteins are expressed in the nucleus of actively growing mammalian cells, their abundance and cellular localization vary greatly in different mouse and human cell types [18,19]. In the present study, we have analyzed whether bifunctional oligonucleotides carrying a tail harboring splicing signals can interfere with the alternative splicing of the Bcl-x pre-mRNA in vitro. We report that a branch site can promote splicing interference when it is part of a 5' or a 3' tail. Moreover, a branched, Y-shaped, 3' tail also interferes with splicing. The interfering activity of a branched tail can be further improved by including splicing signals in the arms of the branch.

## Results

### Splicing interference by 5' tails in bifunctional oligonucleotides

**Figure 1:**
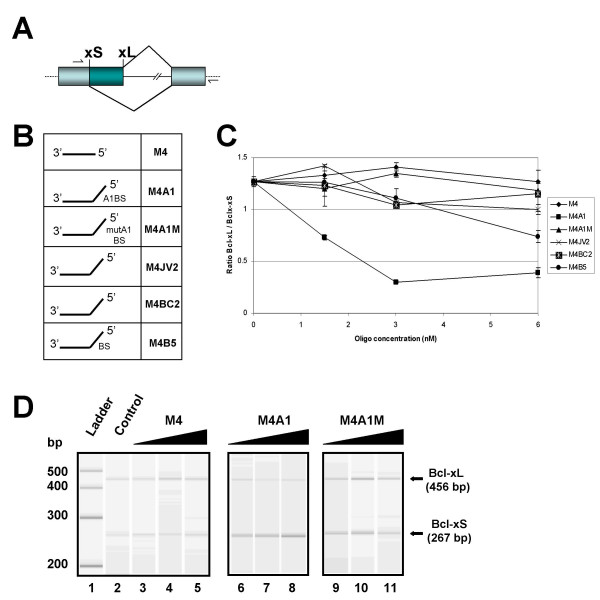
**Activity of bifunctional oligonucleotides carrying a 5' tail**. In vitro splicing assays using a model 964-nt long Bcl-x pre-mRNA incubated in HeLa nuclear extracts in the presence of increasing concentrations of oligonucleotides lacking a tail (M4) or carrying a 5' tail (M4A1, M4A1M, M4JV2, M4BC2 and M4B5). (A) Structure of the human Bcl-x pre-mRNA. Boxes and lines represent exons and introns, respectively. The dashed lines indicate plasmid sequences. The position of the 5' splice sites is shown as well as the position of the primers used in the RT-PCR assay. The portion of the pre-mRNA targeted by hybridization with various oligomers corresponds to nucleotide position -4 to -23, upstream of the Bcl-xL 5' splice junction. (B) Schematic structure of the oligonucleotides. The horizontal portion is complementary to position -4 to -23, upstream of the Bcl-xL 5' splice site. The presence of A1/A2 binding sites (A1BS), mutated A1/A2 binding sites (mutA1BS) or a branch site (BS) is indicated. (C) The RT-PCR assay was carried out on total RNA isolated from splicing reactions performed in triplicate. The RT-PCR products were fractionated by electrophoresis on an Agilent 2100 bioanalyser and the ratio of Bcl-xL/Bcl-xS products was calculated with standard deviations. The results were plotted in a graph that indicates the xL/xS ratio at various concentrations of oligonucleotides. (D) A computer generated image of fluorescent Bcl-x RT-PCR products is shown for the reactions performed in the absence of oligonucleotide (control, lane 2) and in the presence of oligonucleotides M4, M4A1 and M4A1M at concentrations of 1.5, 3.0 and 6.0 nM. The position and size of the Bcl-xL and Bcl-xS products and molecular weight markers are indicated.

**Figure 2:**
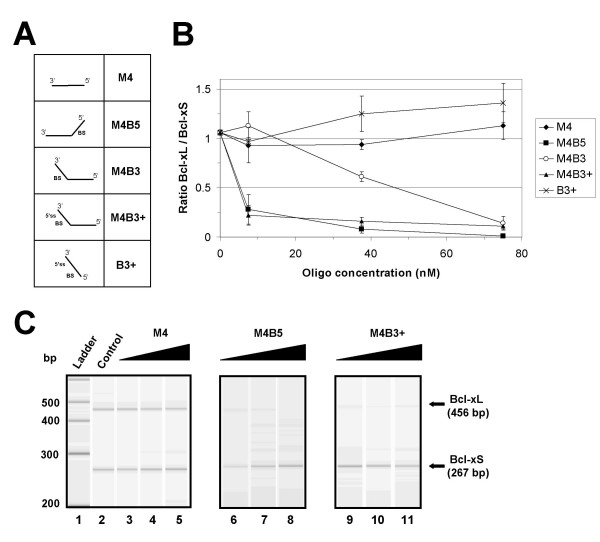
**Activity of bifunctional oligonucleotides containing a 3' tail**. The Bcl-x pre-mRNA was incubated in HeLa extracts in the presence of increasing amounts of oligonucleotides (panel A, BS indicating the presence of a branch site sequence in the tail). Each mixture was tested in triplicate. (B) RT-PCR analysis of splicing mixtures. The ratio of the Bcl-xL and Bcl-xS products was plotted for different concentrations of oligomers. (C) Profiles obtained on the Agilent 2100 bioanalyzer are shown for a control mixture (lane 2) and mixtures incubated with the M4, M4B5 and M4B3+ oligonucleotides at concentrations of 18.75, 37.5 and 75 nM.

We have shown previously that a bifunctional oligonucleotide with a 5' overhang that contains binding sites for the hnRNP A1/A2 proteins strongly inhibits splicing when the oligonucleotide hybridizes immediately upstream of the 5' splice site of Bcl-xL (position -4 to -23). This block increases the production of the alternative Bcl-xS mRNA isoform [17]. Tails made up of poly(U) sequences or containing a purine-rich sequence bound by SR proteins elicited no significant splicing interference. To investigate whether different overhangs could be active, we first tested an oligonucleotide carrying a 21 nt-long tail that contains the sequence of the β-globin branch site (AUAGGCACUGA; the underlined A representing the branchpoint) (M4B5, Fig. 6). A mammalian branch site sequence is recognized initially by the SF1 protein and ultimately by the U2 snRNP during spliceosome assembly [20,21]. RNA oligonucleotides were incubated for 2 hours in a HeLa nuclear extract containing a model ~1 Kb-long human Bcl-x pre-mRNA transcript that contains the 5' splice sites of Bcl-xS and Bcl-xL (Fig. 1A). Total RNA was isolated and a RT-PCR assay was performed to measure the ratio of the Bcl-xS and Bcl-xL amplified products. As observed previously, oligonucleotide M4, which is complementary to positions -4 to -23 upstream of the Bcl-xL donor site but lacks a tail, did not interfere significantly with Bcl-x splicing at any of the concentrations tested (Fig. 1C and 1D). In contrast, oligonucleotide M4A1, which contains a tail with high-affinity binding sites for the A1/A2 proteins, shifted splicing towards Bcl-xS with an IC_50 _of ~1.5 nM (Figs. 1C and 1D). This concentration represents a ~10-fold excess of oligonucleotides relative to the Bcl-x pre-mRNA. The M4A1M oligonucleotide which contains mutations in the A1 binding sites (see Fig. 6) behaved essentially as the tail-less M4 oligomer (Figs. 1C and 1D). Two oligonucleotides containing different control tails of random origins (M4JV2 and M4BC2) also lacked significant activity even at the highest concentration tested (Fig. 6 and Fig. 1C). The oligonucleotide carrying the β-globin branch site sequence as a 5' overhang (M4B5) displayed inhibitory activity, but only at the highest concentration tested (Fig. 1C). Based on this and other experiments (see Fig. 2), the IC_50 _for the M4B5 oligonucleotide was estimated to be in the range of 6 nM. We conclude that while the presence of a tail is not by itself sufficient to elicit splicing interference, a 5' tail containing a branch site can be interfering. Its lower efficiency compared to a tail carrying binding sites for hnRNP A1/A2 proteins may be a characteristic of the tail or may be due to differences in the stability of the oligos. To obtain some assessment of stability, we individually incubated 5' end-labeled oligonucleotides with a similarly labeled control oligonucleotide (B3+) for 0, 30 and 60 minutes in a HeLa extract under splicing conditions. Following incubation, the mixtures were fractionated on gels and the oligonucleotides were quantitated to obtain a stability index for each oligonucleotide. Although 5' end dephosphorylation without degradation will lead to an overestimation of the instability of the oligonucleotides, the assay nevertheless allows the establishment of apparent stability indices that may be useful to a first approximation. Based on this assay (Fig. 5), we conclude that M4A1 and M4A1M display comparable stabilities. M4B5 and M4BC2 also have similar stabilities but are less stable than M4A1, a situation that may explain why M4B5 is less efficient than M4A1. In contrast, the control M4JV2 appears as the least stable oligonucleotide.

### A 3' tail also displays interfering activity

An oligonucleotide carrying a 5' tail bound by hnRNP A1/A2 interferes with splicing in two ways: first, it reduces U1 snRNP binding to the 5' splice site, and second, it interferes with later steps of spliceosome assembly [17]. Because a spliceosome occupies ~25 nt on either side of the 5' splice junction [21], we asked whether a tail emerging 24 nt upstream of the 5' splice junction would be interfering. Thus, we compared the interfering activity of M4B5 with that of an oligonucleotide carrying the β-globin branch site sequence as part of a 3' tail (M4B3; Fig. 6). As can be seen in Fig. 2B, the interfering activity of M4B3 was approximately 7 times inferior to that of M4B5 (IC_50 _of 45 nM for M4B3), despite the fact that the M4B3 appears more stable than M4B5 (Fig. 5). Thus, a branch site tail positioned at the 3' end of a bifunctional oligonucleotide is not as active as a similar tail located at the 5' end.

To try to improve the interfering activity of the 3' tail, we generated oligonucleotide M4B3+, which contains a 5' splice site next to the branch site sequence in the tail of oligonucleotide M4B3 (Fig. 6). The interfering activity of M4B3+ was now indistinguishable from that of M4B5 (Figs. 2B and 2C). It is possible that this 7-fold improvement in activity is in part due to the superior stability of the M4B3+ oligo (a 2.5-fold difference relative to M4B3; see Fig. 5). U1 snRNP binding to the oligonucleotide may provide protection against nucleases. The shift obtained with M4B3+ requires that the tail be linked to the M4 portion of the oligonucleotide since adding a free tail (B3+) to the splicing mixture did not inhibit Bcl-xL mRNA production (Fig. 2B).

### Activity of branched 3' tails

**Figure 3:**
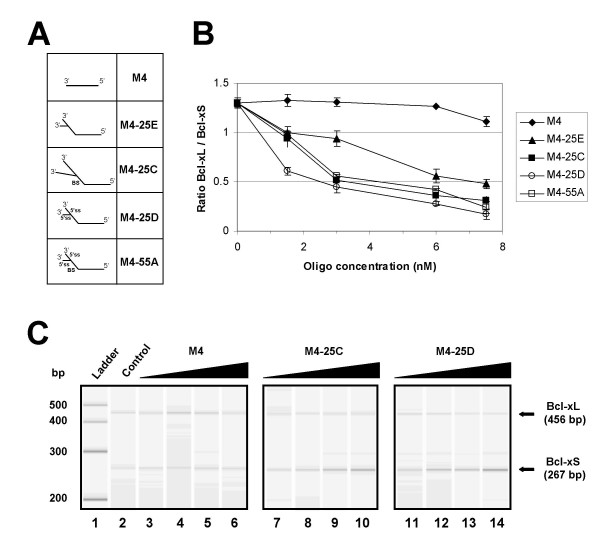
**Activity of bifunctional oligonucleotides with branched 3' tails**. The Bcl-x pre-mRNA was incubated for 2 hours in HeLa extracts in the presence of increasing amounts of oligonucleotides. (A) Schematic structure of the oligonucleotides. The presence in the tail of a branch site (BS) or a 5' splice site (5'ss) is indicated. (B) RT-PCR analysis of splicing mixtures were set up in triplicate. The Bcl-xL/Bcl-xS ratio of amplified products was plotted in the graph relative to the concentration of the oligonucleotides. (C) The profile obtained on the Agilent 2100 bioanalyzer is shown for selected mixtures including a control mixture (lane 2) and mixtures containing oligonucleotides M4, M4-25D and M4-25C at 1.5, 3.0, 6.0 and 7.5 nM. The position and size of the Bcl-x amplified products are shown, as well as the size of molecular weight markers.

Next, we wished to assess whether splicing interference could be improved by using an oligonucleotide that contains a Y-shaped branched structure as part of the 3' tail. A non-hybridizing branched oligonucleotide can inhibit splicing when added at high concentration (5 μM) to a nuclear extract [22], suggesting that the branched structure is bound by splicing factors. A low concentration of a bifunctional oligonucleotide carrying such a branched structure may elicit specific splicing inhibition if positioned near a 5' splice site. Oligonucleotide M4-25E contains three identical 10 nt-long segments of the sequence AAUGUCUGCU. One repeat is linked to the others via a 2'-5' bond (Fig. 6). The 10 nt repeat sequence displays no significant interfering activity when present in a regular 5' tail (M4BC2; Fig. 1C). Notably, incubation of M4-25E promoted a shift toward Bcl-xS production with an IC_50 _of ~5 nM (Fig. 3B), a value comparable to the 6 nM obtained with M4B5 and M4B3+ (Fig. 2). The M4-25E and M4B3+ oligos being of similar stability (Fig. 5), this result suggests that a branched tail provides steric hindrance, possibly by interacting with a branch-specific factor. The inclusion of a branch site sequence at the base of the branched tail (M4-25C, Fig. 6) improved splicing interference (Figs. 3B and 3C; IC_50 _of ~2 nM). Note that this oligonucleotide also contains slightly longer 3' arms but the sequence in the arm is inactive (M4BC2; Fig. 1). The interfering activity of a branched tail was further improved by including a β-globin 5' splice site in the 3' arms of the branch (M4-25D, Fig. 6; Figs. 3B and 3C). With an IC_50 _of 1 nM, the interference level of M4-25D was even slightly superior to that of the 5' tailed oligonucleotide carrying A1/A2 binding sites (Fig. 1; IC_50 _of ~1.5 nM). However, the M4-25C and M4-25D oligos have stability indices 3- to 4-times superior to that of M4A1 (Fig. 5), thereby preventing us to attribute equivalent efficiency based on the design alone. An oligonucleotide with a branched tail carrying both the branch site and the 5' splice site sequences (M4-55A, Fig. 6 and Fig. 3B) was slightly less active than M4-25D but affected splicing in a manner equivalent to the branched oligonucleotide carrying the branch site alone (M4-25C). Because M4-55A was less stable than M4-25C and M4-25D (Fig. 5), the combined presence of both branch site and 5' splice site sequence appears to provide some additional efficacy.

### Role of U1 snRNP in the activity of a 5' splice site tail

**Figure 4:**
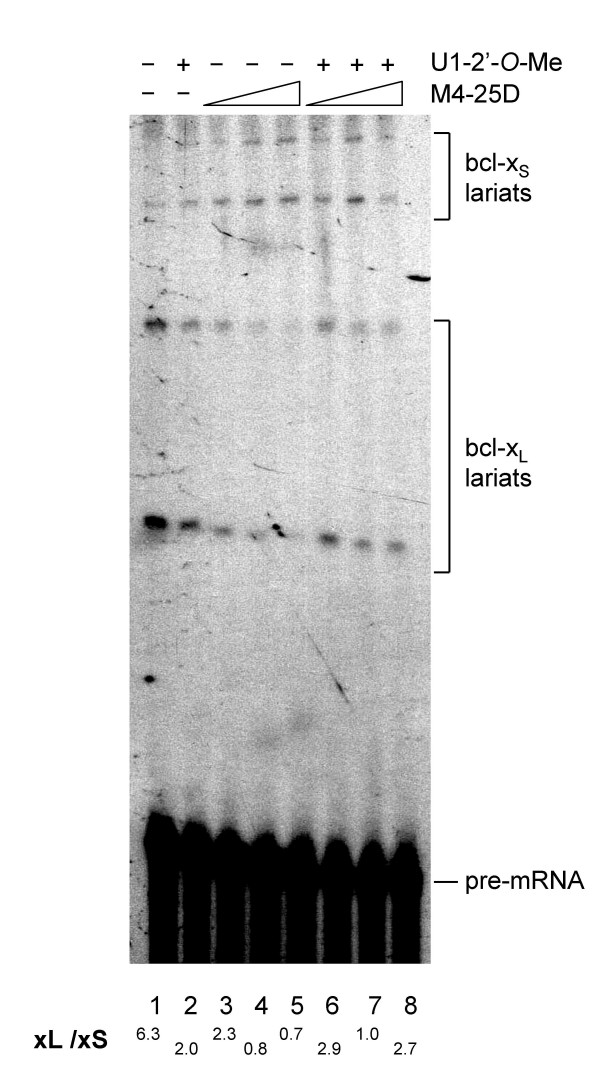
**Importance of U1 snRNP in the activity of the 5' splice site-containing tail**. A uniformly labeled Bcl-x pre-mRNA was incubated for 2 hours in HeLa extracts treated with 0.15 μM of 2'-O-Me oligonucleotides complementary to the 5' end of U1 snRNA. A mock-treated extract was used as control. Increased amounts of the bifunctional oligonucleotide M4-25D were tested (7.4, 37 and 74 nM). Splicing products were fractionated in a 6% denaturing polyacrylamide gel. The position of the lariat splicing products is shown.

**Figure 5:**
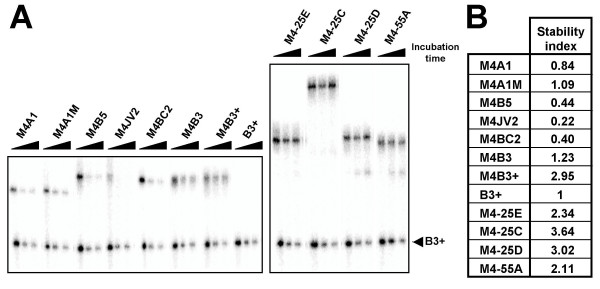
**Stability assessment of the oligos**. (A) Each oligo was 5' end-labeled with ^32^P and mixed with similarly labeled B3+ as an internal control. Following incubation in a HeLa nuclear extract for 0, 30 and 60 minutes, the labeled oligos were fractionated in a 12% denaturing acrylamide gel. (B) The stability indices were tabulated. The apparent stability of each oligo was determined from the results presented in panel A by quantitating the amounts of oligonucleotides remaining after 60 minutes of incubation, and normalizing for the signal of the B3+ control oligonucleotide in each sample. A stability index of 1 is obtained when an oligonucleotide is as stable as B3+. A stability index greater or smaller than 1 indicates that the oligo is more or less stable than B3+, respectively.

We have shown that bifunctional oligos with tails containing branch site or 5' splice site sequences display inhibitory activity when positioned near the Bcl-xL 5' splice site. We wished to ascertain the contribution of U2 and U1 snRNP binding in the respective activity of the branch site and 5' splice site tails. However, this evaluation is complicated by the fact that U1 and U2 are essential splicing factors. Thus, inactivating or depleting U1 or U2 snRNP should block splicing, preventing an assessment of its importance for the activity of the tails. To circumvent this problem, we have tested various concentrations of 2'-O-Me RNA complementary to the 5' end of U1 or U2 snRNA to inactivate U1 or U2 snRNPs [23,24]. Then, we selected a concentration of anti-U1 oligo that had a limited impact on the splicing efficiency of the Bcl-x pre-mRNA, as measured by a conventional splicing assay. Although the selected concentration of the anti-U1 oligo did not affect splicing efficiency, it improved the relative use of the 5' splice site of Bcl-xS such that the xL/xS ratio changed from 6.3 to 2.0 (Fig. 4, lanes 1 and 2, respectively). Next, we asked whether the activity of the interfering tails was affected when the U1 snRNP was targeted in this manner. Notably, the interfering activity of the bifunctional oligo carrying two 5' splice sites in a branched tail (M4-25D) was compromised when we used the extract treated with the 2'-O-Me RNA against the 5' end of U1 snRNA. As shown in Figure 4, in the mock-treated extract, the M4-25D oligo shifted the xL/xS ratio from 6.3 to 2.3 and 0.7 (7.4 and 74 nM of M4-25D, respectively; compare lane 1 with lane 3 and lane 5). In contrast, the xL/xS ratio in the U1-compromised extract went from 2.0 in the control mixture to 2.9 and 2.7 when supplemented with 7.4 or 74 nM of M4-25D oligo, respectively (compare lane 2 with lane 6 and lane 8). Thus, M4-25D almost completely eliminated the production of Bcl-xL lariat products in the mock-treated extract whereas it only had a minor effect in the U1-compromised extract. This result is consistent with the view that the 5' splice site carrying tail of M4-25D is acting by recruiting U1 snRNP. In contrast, similarly targeting U2 snRNP did not affect the activity of a branch site-containing tail (data not shown). This may be because the remaining amount of U2 snRNP is sufficient for the activity of the tail or that another factor is responsible for the activity of the branch site tail.

## Discussion

The current study demonstrates that bifunctional oligonucleotides carrying a variety of tails can efficiently reprogram 5' splice site selection on a model Bcl-x pre-mRNA in vitro. A tailed oligonucleotide provides interaction sites for factors that upon binding will sterically antagonize splice site recognition and/or spliceosome assembly. Our previous work demonstrated that an oligonucleotide carrying a 5' overhang bound by hnRNP A1/A2 proteins and a portion complementary to the sequence immediately upstream of the Bcl-xL 5' splice site compromised the splicing to the Bcl-xL site and improved splicing to the upstream Bcl-xS 5' splice site in vitro and in vivo [17]. Here, we report that a 5' tail containing a branch site sequence also provides significant interference. It is not clear why the branch site-containing oligonucleotide is less active than the A1/A2-bound oligonucleotide. The oligonucleotide may fold in a way that reduces its hybridization to the target site or compromises the binding of factors to the branch site sequence. Alternatively, the binding of splicing factors may be less efficient or less obstructive than when the tail is recruiting hnRNP A1/A2 proteins. Finally, the lower stability of the branch site oligonucleotide may also contribute to this difference with the oligonucleotide containing A1/A2 binding sites.

A branch site sequence presented in a 3' tail was considerably less active than when part of a 5' tail, suggesting that the interfering potential of the tail decreases when its distance relative to the 5' splice site of Bcl-xL increases. Alternatively, the branch site factor may bind less efficiently in this configuration. In addition, we have observed that a 3' tail containing a Y-shaped branched structure can interfere with splicing. Branched structures carrying 2'-5' linkage are recognized by factors that promote debranching [25]. Gel-shift assays indicated that the branched tail of oligonucleotide M4-25E was stably bound by nuclear factors, whereas no stable binding was observed with the individual arms of the branched tail (data not shown). Although the identity of the factor(s) that binds to the branch in these assays remains unknown, our results indicate that positioning a branch 24 nt upstream of a 5' splice site represents a strong impediment to splicing. Finally, a stronger splicing interference was obtained when the branched tail included a β-globin branch site or 5' splice site. Thus, combining a branched core with spliceosomal factor recognition elements such as a branch site or a 5' splice site improved splicing inhibition. A branched oligonucleotide with a 5' splice site sequence in each of the 3' extensions was the most efficient branched oligomer tested with an IC_50 _of 1 nM.

Our results clearly demonstrate that antisense oligonucleotides containing sterically interfering overhangs can modulate 5' splice site selection. In addition to a 5' tail carrying binding sites for hnRNP A1/A2, tails of completely different configurations worked efficiently, thereby providing flexibility in the choice of an interfering tail. The hnRNP A1 and A2 proteins are expressed in the majority of transformed cells but are not abundantly expressed in many normal human tissues [18]. Thus, tails with binding sites for constitutively expressed factors (like 5' splice site, branch site and branch binding factors) may be more suitable if the intention is to reprogram splicing in normal cells.

Interfering oligonucleotides are active at low nanomolar concentrations in vitro. We have shown that interfering bifunctional 2'-O-Me oligonucleotides are also active in vivo at concentrations below 50 nM [17]. Such high potency makes them attractive alternatives to current RNAi approaches which use short double-stranded RNAs that, in some instances, can elicit an unwanted interferon response [26-29]. An additional advantage relative to RNAi-based approaches is that bifunctional oligomers can be used to repress the production of one isoform while simultaneously increasing the production of another isoform. This may be desirable when the purpose of the intervention is to alter the ratio of spliced isoforms displaying different, sometimes antagonistic activities, as is the case with the apoptotic regulator Bcl-x.

Different strategies using tailed oligomers have recently been described as means of reprogramming alternative splicing decisions. Using a tail carrying binding sites for SR proteins, Skordis and collaborators have shown that such a tail can recapitulate the activity of an exon enhancer element by stimulating the inclusion of exon 7 of SMN2 in vitro and in vivo [30]. Likewise, PNA-peptide oligomers carrying 5 to 15 arginine-serine residues stimulated the in vitro inclusion of two weak exons (BRCA1 exon 18 and SMN2 exon 7) [31]. In contrast to the two preceding strategies, our approach relies on using a tail to elicit splicing inhibition. It is possible that in all cases, the hybridization of the oligonucleotide to exon sequences may also repress mRNA translation, a situation that would antagonize the impact of a splicing enhancing tail, but would further contribute towards abrogating the expression of a specific splice isoform when a splicing inhibitory tail is used. For the moment however, an increase in the arsenal of strategies that can reprogram splicing provides greater flexibility in the choice of approaches to alter splicing decisions in genes that affect human diseases.

## Conclusion

Alternative pre-mRNA splicing is relevant to a large variety of human diseases including cancer. As a means of reprogramming splice site selection, we have used bifunctional oligos that hybridize to exon sequences and contain a tail designed to interfere with the use of a nearby 5' splice site. We have tested the activity of different tail architectures and found that tails containing a branched structure, a branch site or a 5' splice site bound by U1 snRNP can reprogram in vitro splicing at low nanomolar concentrations. Thus, a variety of tail structures bound by different factors may be used to reprogram 5' splice site selection.

## Methods

### Synthesis and purification of oligonucleotides

Linear antisense RNA oligonucleotide constructs were synthesized on an ABI 381A DNA synthesizer using standard silyl-phosphoramidite chemistry [32]. Conversely, branched RNAs were assembled using a well-established convergent solid-phase methodology [33]. The conserved adenosine branchpoint was introduced *via *the adenosine bis-phosphoramidite under dilute conditions (0.03 M) [34,35]. To afford maximal branching, high-loading CPG was utilized [33,35]. Additionally, antisense molecules less than 40 nucleotides in length were constructed on a 500 Å controlled-pore glass (CPG) support, whereas longer oligonucleotides (> 40-nt) were assembled using a 1000 Å CPG pore size to prevent steric clashing between the growing oligonucleotide strands. The antisense oligonucleotides were designed to contain a 20-nucleotide portion, which was complementary to the region directly upstream (positions -4 to -23) of the Bcl-xL 5'-splice site in the second exon of Bcl-x.

**Figure 6:**
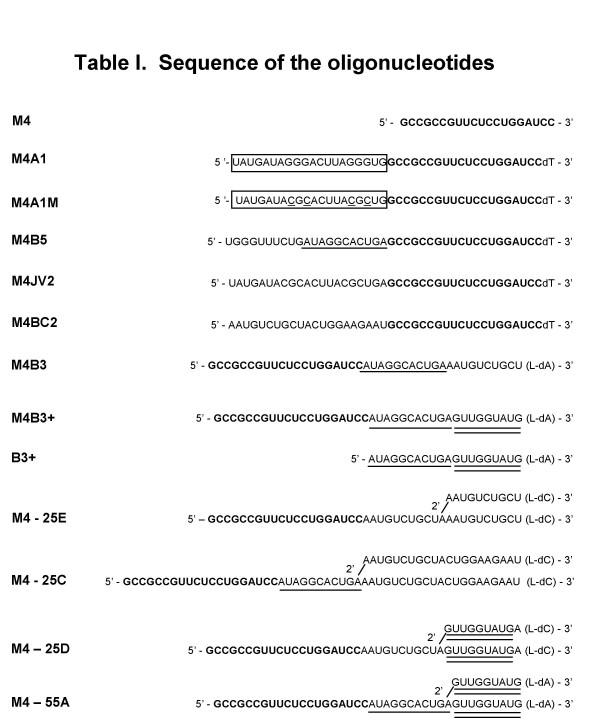
Sequence of the oligonucleotides. The sequence in bold indicates complementarity to the region 4 to 23 nt upstream of the Bcl-xL 5' splice site. Boxed sequences contain the hnRNP A1 high-affinity binding sites UAGGGA and UAGGGU. The mutations in M4A1M are underlined. The underlined sequence in M4B5, M4B3, B3, and M4-25C corresponds to a human β-globin branch site sequence, while the double underlined sequence represents a β-globin 5' splice site sequence.

In an effort to stabilize the oligomers against exonucleases present in HeLa extracts, dT or unnatural L-deoxynucleotides (L-dC and L-dA) were introduced at the 3'-termini of the molecules (see Fig. 6) using the appropriate nucleoside loaded CPG [36]. Oligonucleotides were deprotected under standard conditions along with an ensuing treatment with the desilylation reagent, triethylamine hydrofluoride (TREAT-HF) to remove the 2'-tert-butyldimethylsilyl (TBDMS) protecting group. Furthermore, the molecules were analyzed and purified by denaturing polyacrylamide gel electrophoresis (PAGE) (12% acrylamide, 7 M urea), desalted by size-exclusion chromatography (Sephadex G-25) and their nucleotide composition confirmed by MALDI-TOF-MS [33]. Analysis of the oligonucleotide products by denaturing PAGE revealed that all of the compounds were synthesized with high efficiency.

### Splicing and RT-PCR assays

The Bcl-x template used for in vitro transcription was made from the pBc5/3 plasmid by PCR amplification [37]. The PCR products were purified and the Bcl-x pre-mRNA was synthesized using T3 RNA polymerase (USB) in the presence of cap analog. The Bcl-x pre-mRNA contained the last 331 nt of exon 2 followed by 434 nt of intron 2 and 137 nt of exon 3. The pre-mRNA was gel-purified as described [38] and quantified by spectroscopic analysis or using an Agilent 2100 bioanalyzer station. Following the addition of the interfering oligonucleotides in a HeLa nuclear extract [39], two fmoles (150 pM) of the Bcl-x pre-mRNA were added and the mixture was incubated for 2 hours under standard splicing conditions [40]. All splicing reactions were carried out in triplicate. Total RNA was extracted and purified. The reverse transcription step was performed at 37°C for 1 h using Omniscript (Qiagen) with 0.75 μM of the bc2b primer (CGCTCTAGAACTAGTGGATC). The reaction was followed by PCR using the following procedure: 95°C for 3 min; 35 cycles at 93°C for 15 sec, 60°C for 30 sec, 72°C for 30 sec; and a final extension at 72°C for 3 min. Products were amplified using 0.3 μM each of primers BX2 (TCATTTCCGACTGAAGAGTGA) and BX3 (ATGGCAGCAGTAAAGCAAGCG). The amplified products were fractionated and quantitated using the Agilent 2100 bioanalyzer.

For conventional splicing assays, a uniformly ^32^P-labeled transcript was produced from T3 RNA polymerase transcription of the PCR product derived from pBc5/3. After incubation in HeLa extracts, the RNA products were fractionated in a 6% acrylamide/7 M urea gel and the splicing products were quantified on a PhosphorImager. Splicing assays performed in the presence of anti-U1 (CCUGCCAGGUAAGUA) and anti-U2 (AGAACAGAUACUACACUUGA) 2'-O-Me oligos contained 0.15 μM of the respective oligos.

For stability assays in nuclear extracts, oligos were 5' end labeled with T4 polynucleotide kinase and ^32^P-γ-ATP. A quantity of each oligo (8.0 nM) was incubated with equivalent amounts of the B3+ oligo. Following fractionation in a denaturing gel, the cpm values of each oligo in each sample were counted directly using an InstantImager (Canberra-Packard). The ratio cpm_60 min_/cpm_0 min _(normalized for the intensity of the control oligo) was calculated as the stability index. Values above and below 1.0 respectively indicate a stability higher and lower than the control B3+ oligo.

## List of abbreviations

hnRNP: heterologous nuclear ribonucleoprotein particle; PCR: polymerase chain reaction; snRNP, small nuclear ribonucleoprotein; 2'-O-Me; 2'-O-Methyl.

## Authors' contributions

DG, DG and JV performed splicing experiments. SC synthesized the oligos. RK, SAE, MJD and BC supervised the project. MJD and BC drafted the final manuscript. All authors approved the final manuscript.

## References

[B1] Johnson JM, Castle J, Garrett-Engele P, Kan Z, Loerch PM, Armour CD, Santos R, Schadt EE, Stoughton R, Shoemaker DD (2003). Genome-wide survey of human alternative pre-mRNA splicing with exon junction microarrays. Science.

[B2] Wu JY, Tang H, Havlioglu N (2003). Alternative pre-mRNA splicing and regulation of programmed cell death. Prog Mol Subcell Biol.

[B3] Jiang ZH, Wu JY (1999). Alternative splicing and programmed cell death. Proc Soc Exp Biol Med.

[B4] Boise LH, Gonzalez-Garcia M, Postema CE, Ding L, Lindsten T, Turka LA, Mao X, Nunez G, Thompson CB (1993). bcl-x, a bcl-2-related gene that functions as a dominant regulator of apoptotic cell death. Cell.

[B5] Tu Y, Renner S, Xu F, Fleishman A, Taylor J, Weisz J, Vescio R, Rettig M, Berenson J, Krajewski S, Reed JC, Lichtenstein A (1998). BCL-X expression in multiple myeloma: possible indicator of chemoresistance. Cancer Res.

[B6] Reeve JG, Xiong J, Morgan J, Bleehen NM (1996). Expression of apoptosis-regulatory genes in lung tumour cell lines: relationship to p53 expression and relevance to acquired drug resistance. Br J Cancer.

[B7] Olopade OI, Adeyanju MO, Safa AR, Hagos F, Mick R, Thompson CB, Recant WM (1997). Overexpression of BCL-x protein in primary breast cancer is associated with high tumor grade and nodal metastases. Cancer J Sci Am.

[B8] Krajewska M, Krajewski S, Epstein JI, Shabaik A, Sauvageot J, Song K, Kitada S, Reed JC (1996). Immunohistochemical analysis of bcl-2, bax, bcl-X, and mcl-1 expression in prostate cancers. Am J Pathol.

[B9] Clarke MF, Apel IJ, Benedict MA, Eipers PG, Sumantran V, Gonzalez-Garcia M, Doedens M, Fukunaga N, Davidson B, Dick JE (1995). A recombinant bcl-x s adenovirus selectively induces apoptosis in cancer cells but not in normal bone marrow cells. Proc Natl Acad Sci U S A.

[B10] Ealovega MW, McGinnis PK, Sumantran VN, Clarke MF, Wicha MS (1996). bcl-xs gene therapy induces apoptosis of human mammary tumors in nude mice. Cancer Res.

[B11] Mercatante DR, Bortner CD, Cidlowski JA, Kole R (2001). Modification of alternative splicing of Bcl-x pre-mRNA in prostate and breast cancer cells. analysis of apoptosis and cell death. J Biol Chem.

[B12] Taylor JK, Zhang QQ, Monia BP, Marcusson EG, Dean NM (1999). Inhibition of Bcl-xL expression sensitizes normal human keratinocytes and epithelial cells to apoptotic stimuli. Oncogene.

[B13] Sumantran VN, Ealovega MW, Nunez G, Clarke MF, Wicha MS (1995). Overexpression of Bcl-XS sensitizes MCF-7 cells to chemotherapy-induced apoptosis. Cancer Res.

[B14] Sazani P, Kole R (2003). Modulation of alternative splicing by antisense oligonucleotides. Prog Mol Subcell Biol.

[B15] Sazani P, Gemignani F, Kang SH, Maier MA, Manoharan M, Persmark M, Bortner D, Kole R (2002). Systemically delivered antisense oligomers upregulate gene expression in mouse tissues. Nat Biotechnol.

[B16] Scaffidi P, Misteli T (2005). Reversal of the cellular phenotype in the premature aging disease Hutchinson-Gilford progeria syndrome. Nat Med.

[B17] Villemaire J, Dion I, Elela SA, Chabot B (2003). Reprogramming alternative pre-messenger RNA splicing through the use of protein-binding antisense oligonucleotides. J Biol Chem.

[B18] Patry C, Bouchard L, Labrecque P, Gendron D, Lemieux B, Toutant J, Lapointe E, Wellinger R, Chabot B (2003). Small interfering RNA-mediated reduction in heterogeneous nuclear ribonucleoparticule A1/A2 proteins induces apoptosis in human cancer cells but not in normal mortal cell lines. Cancer Res.

[B19] Kamma H, Portman DS, Dreyfuss G (1995). Cell type-specific expression of hnRNP proteins. Exp Cell Res.

[B20] Kramer A (1992). Purification of splicing factor SF1, a heat-stable protein that functions in the assembly of a presplicing complex. Mol Cell Biol.

[B21] Chabot B, Steitz JA (1987). Multiple interactions between the splicing substrate and small nuclear ribonucleoproteins in spliceosomes. Mol Cell Biol.

[B22] Carriero S, Damha MJ (2003). Inhibition of pre-mRNA splicing by synthetic branched nucleic acids. Nucleic Acids Res.

[B23] Barabino SM, Blencowe BJ, Ryder U, Sproat BS, Lamond AI (1990). Targeted snRNP depletion reveals an additional role for mammalian U1 snRNP in spliceosome assembly. Cell.

[B24] Barabino SM, Sproat BS, Ryder U, Blencowe BJ, Lamond AI (1989). Mapping U2 snRNP--pre-mRNA interactions using biotinylated oligonucleotides made of 2'-OMe RNA. EMBO J.

[B25] Ruskin B, Green MR (1985). An RNA processing activity that debranches RNA lariats. Science.

[B26] Pebernard S, Iggo RD (2004). Determinants of interferon-stimulated gene induction by RNAi vectors. Differentiation.

[B27] Sledz CA, Holko M, de Veer MJ, Silverman RH, Williams BR (2003). Activation of the interferon system by short-interfering RNAs. Nat Cell Biol.

[B28] Persengiev SP, Zhu X, Green MR (2004). Nonspecific, concentration-dependent stimulation and repression of mammalian gene expression by small interfering RNAs (siRNAs). RNA.

[B29] Rossi F, Labourier E, Forne T, Divita G, Derancourt J, Riou JF, Antoine E, Cathala G, Brunel C, Tazi J (1996). Specific phosphorylation of SR proteins by mammalian DNA topoisomerase I. Nature.

[B30] Skordis LA, Dunckley MG, Yue B, Eperon IC, Muntoni F (2003). Bifunctional antisense oligonucleotides provide a trans-acting splicing enhancer that stimulates SMN2 gene expression in patient fibroblasts. Proc Natl Acad Sci U S A.

[B31] Cartegni L, Krainer AR (2003). Correction of disease-associated exon skipping by synthetic exon-specific activators. Nat Struct Biol.

[B32] Usman N, Ogilvie KK, Jiang MY, Cedergren RG (1987). Automated chemical synthesis of long oligoribonucleotides using 2'-O-silylated ribonucleoside 3'-O-phosphoramidites on a controlled-pore glass support: Synthesis of a 43-nucleotide sequence similar to the 3'-half molecule of Escherichia coli formylmethionine tRNA.. J Am Chem Soc.

[B33] Carriero S, Damha MJ, Beaucage SL, Glick GD, Bergstrom DE and Jones RA (2002). Solid-Phase Synthesis of Branched Oligonucleotides.. Current Protocols in Nucleic Acids Chemistry.

[B34] Damha MJ, Zabarylo SV (1989). Automated Solid-Phase Synthesis of Branched Oligonucleotides. Tetrahedron Letters.

[B35] Damha MJ, Ganeshan K, Hudson RH, Zabarylo SV (1992). Solid-phase synthesis of branched oligoribonucleotides related to messenger RNA splicing intermediates. Nucleic Acids Res.

[B36] Damha MJ, Giannaris PA, Marfey P (1994). Antisense L/D-oligodeoxynucleotide chimeras: nuclease stability, base-pairing properties, and activity at directing ribonuclease H. Biochemistry.

[B37] Garneau D, Revil T, Fisette JF, Chabot B (2005). hnRNP F/H proteins modulate the alternative splicing of the apoptotic mediator Bcl-x. J Biol Chem.

[B38] Chabot B, Higgins SJHBD (1994). Synthesis and purification of RNA substrates. RNA processing, A practical approach.

[B39] Dignam JD, Lebovitz RM, Roeder RG (1983). Accurate transcription initiation by RNA polymerase II in a soluble extract from isolated mammalian nuclei. Nucleic Acids Res.

[B40] Krainer AR, Maniatis T, Ruskin B, Green MR (1984). Normal and mutant human beta-globin pre-mRNAs are faithfully and efficiently spliced in vitro. Cell.

